# Peritoneal expression of matrilysin helps identify early post-operative recurrence of colorectal cancer

**DOI:** 10.18632/oncotarget.2830

**Published:** 2015-01-21

**Authors:** Giuseppe S. Sica, Cristina Fiorani, Carmine Stolfi, Giovanni Monteleone, Eleonora Candi, Ivano Amelio, Valeria Catani, Simone Sibio, Andrea Divizia, Giorgia Tema, Edoardo Iaculli, Achille L. Gaspari

**Affiliations:** ^1^ Department of Experimental Medicine and Surgery, Tor Vergata University, Rome, Italy; ^2^ European Society Degenerative Disease (ESDD). www.esdd.it; ^3^ Medical Research Council, Toxicology Unit, Leicester, UK

## Abstract

Recurrence of colorectal cancer (CRC) following a potentially curative resection is a challenging clinical problem. Matrix metalloproteinase-7 (MMP-7) is over-expressed by CRC cells and supposed to play a major role in CRC cell diffusion and metastasis. MMP-7 RNA expression was assessed by real-time PCR using specific primers in peritoneal washing fluid obtained during surgical procedure. After surgery, patients underwent a regular follow up for assessing recurrence. transcripts for MMP-7 were detected in 31/57 samples (54%). Patients were followed-up (range 20–48 months) for recurrence prevention. Recurrence was diagnosed in 6 out of 55 patients (11%) and two patients eventually died because of this. Notably, all the six patients who had relapsed were positive for MMP-7. Sensitivity and specificity of the test were 100% and 49% respectively. Data from patients have also been corroborated by computational approaches. Public available coloncarcinoma datasets have been employed to confirm MMP7 clinical impact on the disease. Interestingly, MMP-7 expression appeared correlated to Tgfb-1, and correlation of the two factors represented a poor prognostic factor. This study proposes positivity of MMP-7 in peritoneal cavity as a novel biomarker for predicting disease recurrence in patients with CRC.

## INTRODUCTION

Colorectal cancer (CRC) is the second most commonly diagnosed solid malignancy in women and the third most common in men worldwide, accounting for over 600,000 deaths in 2008 [1–9]. In the United States, CRC is the second leading cause of cancer death accounting for approximately 9% of deaths related to cancer overall [10]. Adenocarcinoma represents the vast majority of CRC and 70% of all malignant tumors of gastrointestinal tract. One in three people who develop CRC will die because of it and approximately 20% will develop metastatic CRC [11–13].

The prognosis of CRC patients depends on the histologic type, cell differentiation, TNM stage and the patient's chances of being subjected to radical surgery. For patients who presents with macroscopic peritoneal metastases, treatment options are limited. Hypertermic intraperitoneal chemiotherapy (HIPEC) associated to aggressive tumor debulking surgery has been proposed in subset of patients [14–21]. Detection of tumor cells within the peritoneal cavity at the time of surgery has been proposed to identify patients with poorer outcome overall. Positive free cancer cells in the peritoneal cavity (IFTC) can be detected in a variable percentage of CRC patients at the time of surgery [22, 23]. The current available methods for IFTC identification include conventional cytology, immunohistochemistry or real-time PCR for the cytokeratin 20 (KRT-20) or carcinoembryonic antigen (CEA). However the presence of IFTC is not currently used as a prognostic factor due to the heterogeneity in sampling and analysis among different studies. This is likely due to the lack of a standard method that allows IFTC identification with sufficient accuracy.

Several factors have been implicated in CRC prognosis [24–31], including cell cycle/apoptotic regulators [32–49], cellular motility factors [50–61] and metabolic enzymes [62–74]. For example, mutations of the tumour suppression factors p53 [75–78] have been described in about 40% to 50% of colorectal carcinomas. LOH of the short arm of chromosome 17 is also found in most of these tumors and are associated with aggressive tumors. [79–83] Besides the loss of function of wt p53 [54, 84–94], mutant p53 retains additional ability to promote tumorigenicity and tumor progression, giving rise to what it has been defined gain-of-function of mp53 [78, 95]. Metallopeptidases are also been involved in tumourigenesis, in particular in late stages such as invasion and metastasis [96–98], underlining their importance for recurrence and consequence recovery from the disease. In CRC [99–104], metalloproteinases are secreted as inactive enzyme and are activated extracellularly. These enzymes, in particular MMP−1, −2, −3. −7, −9, −13, have been demonstrated to be expressed in human colorectal cancers. Often, the degree of over expression of some of them has been positively associated with stage of disease and/or poor prognosis. Polymorphisms in promoter regions of MMP genes might be related to the susceptibility of digestive cancers, with a role in cancer development for MMP1 and MMP7, and a role of protection against cancer for MMP2 and MMP9 [105]. Accordingly, high level of MMP7 has been hystochemically detected in CRC and revealed in serum of CRC patients [106]. It is interesting to note that it is still an unresolved point whether MMPs are produced by cells surrounding a tumor or by the colorectal cancer cells themselves. In our study the real time PCR for a matrixmetalloproteinase 7 (MMP-7) is employed. MMP are a family of zinc-dependent endopeptidases with proteolytic activity. MMP-7 or matrilysinis is not expressed by normal colonic epithelial cells, but it is highly expressed at high levels by colonic neoplastic cells. In this particular case, the presence of a mutation in the APC gene causes accumulation of beta-Catenin/TCF complex in the nucleus, and as a consequence, up regulation of MMP-7 expression. MMP-7 targets laminina-5/laminina-332 (LN5), an important component of the basement membrane and epithelial cell adhesion that guarantees the formation of hemidesmosomes. Due to this action, MMP-7 is involved in the degradation of extracellular matrix (ECM), adaptation of tumour microenvironment [107–110] and thereby promoting the process of invasion and metastasis. Highlighting the expression of this enzyme we are able to identify, among all the IFTC, those that have greater capacity for engraftment [84, 111, 112]. In a recent study was demonstrated the diagnostic value of serum MMP-7 levels in bladder cancer [113, 114].

This study was aimed at determining whether MMP-7 is detectable in the peritoneal cavity of CRC patients undergoing potentially curative resection and assessing whether MMP-7 positively marks patients that eventually develop CRC recurrence.

## RESULTS

### Peritoneal expression of mmp7 in colorectal cancer

**Figure 1:**
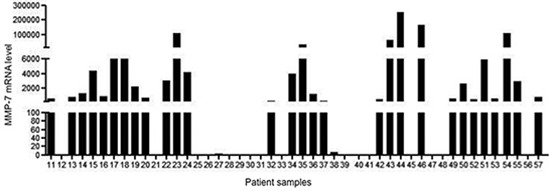
Expression of MMP-7 RNA transcripts in 47 peritoneal washing samples taken from 47 patients who had undergone surgery for colorectal cancer

Sixty-seven peritoneal washing were performed in 67 patients who underwent surgery for colon and intraperitoneal rectal cancer. The first 10 lavages were needed to set the methodology whereas the subsequent 47 samples were the object of the study and used to assess MMP-7 expression by real-time PCR. MMP-7 transcripts were detected in 31/57 samples (54%) (Fig. 1)

At surgery, curative resection was achieved in 56 patients. In one patient, the primary tumor was not excised because of peritoneal carcinomatosis and liver metastases. Apart from this latter case, synchronous liver metastases were present in another three patients. One patient showed peritoneal dissemination at surgery and underwent local peritonectomy. Patients demographics, tumor location, histological type, tumor grading and stage are reported in tab 1.

**Table 1:** Patients demographics and tumor characteristics

Tumor type and demographics			n.			%		
**Gender**								
M			19			33		
F			38			67		
**Age (mean 68 [range 37–91])**								
> 60			43			75		
< 60			14			25		
**Localization**								
right			22			39		
transverse			2			3		
left			25			44		
rectum			8			14		
**Histology**								
adenocarcinoma			52			91		
mucinous			5			9		
**Grading**	**Stage (UICC)**	**(Dukes)**						
G1	I	A	7	18	1	12	32	2
G2	II	B (1–2)	33	20	11 26	58	35	19 46
G2–G3	III	C (1–2)	12	15	0 15	21	26	0 26
G3	IV	D	5	4	4	9	7	7

Patients were followed up for a mean period of 34 months (range 20–48 months). Two patients were lost at the follow up. Six of 56 patients undergone colonic resection, eventually presented with tumor recurrence (10.7%). Type of recurrence are presented in tab 2. Six patients died during follow up:one is the patients found inoperable; one within the patients with synchronous metastases; two patients died because of their relapse; the other two deaths occurred because of surgical complication in one case and because of ageing in the second patient. Deaths are also reported in tab 2.

**Table 2:** Cause of death and type of recurrence

**Death:**	
**Inoperable (liver mets + carcinomatosis)**	1
**Recurrence**	2
Bone and lungmets	
Local recurrence	
**Syncronous liver mets**	1
**Non cancerous death**	2
**Recurrence:**	
Peritoneal carcinomatosis	1
Local recurrence	2
Distant metastasis	3

Within the 3 patients that had liver metastases pre-operatively; 2 patients who had developed recurrence (1 to lung and bones and 1 local at anastomotic site).

Notably, MMP-7 was expressed in all the 6 patients who had relapsed, thus showing a sensitivity of 100%, whereas the specificity of the method is 49%.

### MMP7 in colon carcinoma

**Figure 2:**
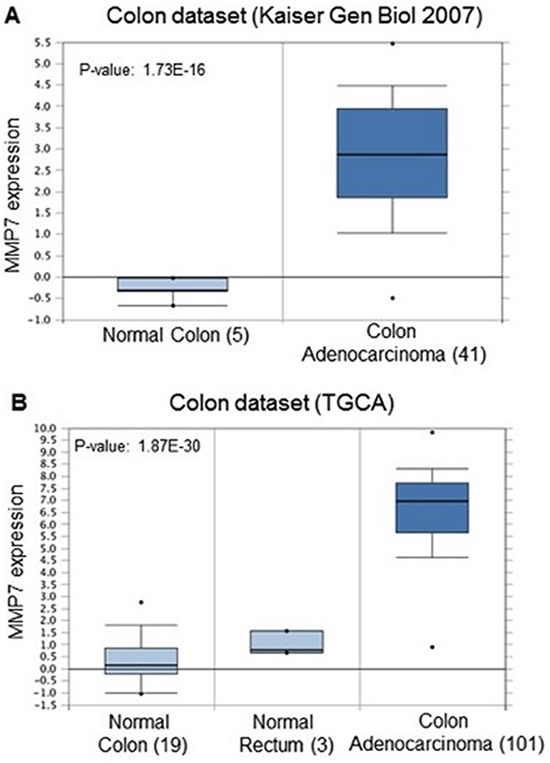
**(A, B)** Comparison of MMP7 expression level in colon carcinomas and Normal colon or rectum epithelia in different datasets. MMP7 mRNA levels are upregulated in malignant lesions compared to normal counterparts. Numbers in brackets indicate number of sample analyzed in each group. *P* value: 1.73E-16 (A) and 1.87E-30 (B) oncomine.org.

**Figure 3:**
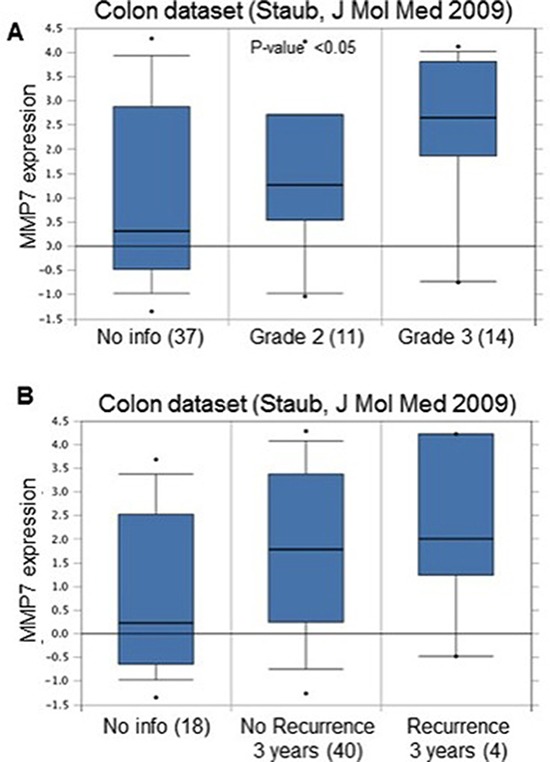
**(A)** MMP7 is upregulated in high-grade colon carcinomas. Grade 3 tumours showed increased mRNA levels compared to Grade 2. *P*-value 0.05. Numbers in brackets indicate number of sample analyzed in each group. No info indicates samples without tumour grade information. **(B)** MMP7 is upregulated in colon carcinoma patients with recurrence within the first 3 years. *P*-value 0.05. Numbers in brackets indicate number of sample analyzed in each group. No info indicates samples without recurrence information. oncomine.org.

**Figure 4:**
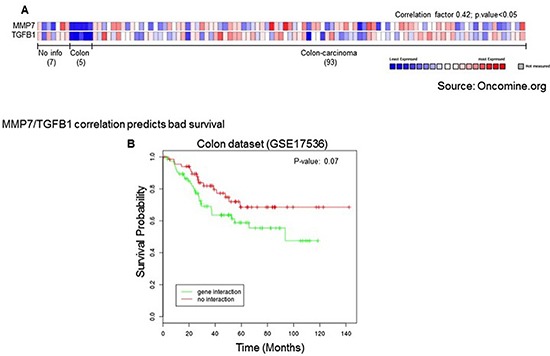
**(A)** Coexpression analysis revealed direct correlation between MMP7 and TGFb1. Correlation factor 0.42, *p* value < 0.05 oncomine.org. **(B) 4** Positive MMP7/TGFB1 correlation represents a prognostic factor for bad patient survival. Patient survival estimation of MMP7/TGFB1 positive correlation group (“gene interaction”) compared to negative or absent correlation group (“no interaction”). *P* value = 0.07.

In order to identify a potential clinical relevance for MMP7 in human coloncarcinoma we screened publicity available human adenocarcinoma datasets for different hystopathological and prognostic parameters. Basic expression analysis of MMP7 revealed that its mRNA levels are specifically upregulated in colon malignancies when compared to normal colon epithelial tissues. Colon carcinomas showed an upregulation between 3.8 and 7.1 depending on the dataset analyzed and the variety of the pool of samples (Fig. 2). When datasets with clinical parameters were selected for the analysis, stratification for tumour grade or recurrence further identified MMP7 selective expression. Indeed MMP7 expression levels resulted enriched in tumour with high grade (tumour grade 3) and in patients, which encountered recurrence within 3 years from the primary tumour onset (Fig. 3). This analysis highlighted the efficacy of MMP7 to specifically subselect tumours with higher aggressiveness, suggesting a potential role of negative prognostic marker. To further characterize MMP7 positive expressing colon carcinomas and to go insight a potential mechanism for MMP7 function in these tumours, we performed an expression analysis for MMP73 and putative coexpressed factors. Among the top rated genes we identified transforming growth factor beta 1 (TGFb1) as a statistically significant coexpressed factors with MMP7 (Correlation factors 0.4) (Fig. 4A). Notably 5 normal colon samples do not show expression neither MMP7 nor TGFb1. TGF-beta 1 plays a role as a tumor suppressor in early disease [115–122] and has pro-oncogenic effects as well as drug-resistance [123–136] in advanced tumor stage [121, 123, 137, 138], in particular in metastasis process [139–143]. Therefore MMP7/TGFb1 coexpression in advanced coloncarcinomas would suggest a potential synergistic negative impact of these two factors on the clinical outcome. To assess the impact of MMP7/TGFb1 on patient survival we performed a computation analysis, stratifying the samples in two groups: samples where MMP7 and TGFb1 positively correlate (“gene interaction”) and samples where they do not correlate (“no gene interaction”). Computation estimation of Kaplan-Maier in these two subgroups revealed that coexpression of MMP7 and TGFb1 negatively affected survival outcome of colon carcinoma patients (Fig. 4B)

## DISCUSSION

The TNM system is of basic prognostic value in solid tumor. Peritoneal carcinomatosis means metastatic disease, and curative resection is only seldom possible. The role of peritoneal cytology in gynecology is well defined, and it is formally incorporated into the TNM staging for ovarian and endometrial cancer [144–153]. Positive peritoneal washing represent an independent prognostic factor for poor survival also in patients with cancer of the gastrointestinal tract [154]. Peritoneal lavage in search for IFTC can be part of the staging protocol in case for pancreatic adenocarcinoma, and for cancer of the cardia or stomach. [155–158] In CRC, the occurrence of isolated tumor cells in the peritoneal washings could be correlated with a worse prognosis, even in early stages. Furthermore, if it is clear that the serosal cancer cells can implant within the peritoneum [159], it has been shown that they have the potential to enter both the lymphatics and the systemic circulation, thus showing a more aggressive and motile phenotype [107, 160–163].

Therefore, the presence of IFTC could serve as prognostic marker to guide adjuvant therapy [164].

Cytopathology, immunocytochemistry (ICC) and polymerase chain reaction (PCR) are the different methods in use for defining the presence of neoplastic cells in the peritoneum at the time of colorectal cancer surgery. The wide range in positive lavage across studies illustrates the difficulty in comparing studies with different methods of detection of positive lavage fluid. The overall mean rate of positive lavage is 13.7% [165]. Conventional staining (Papanicolau, PAS, Giemsa) techniques are relatively inexpensive and do not requires preservation of RNA. ICC, also a histological staining technique, appears to result in a far greater yield of IFTC thancytopathology [166–168]. In fact, in cytological examinations of peritoneal fluid, the distinction of mesothelial cells and tumor cells might be difficult. It is crucial to detect epithelial cells; however, ICC is subjective and depends on the strength of cellular staining [169, 170]. PCR-based methods are suitable for detecting minute quantities of intraperitoneal free cancer cells but, as they detect DNA and not viable cells, there is a problem in differentiate cancerous cells from non-malignant cells or cellular debris [171]. The most commonly used markers, specific for cancer cells, are the CEA and the CK-20 for gastric and colorectal cancer [155, 156], and the C1P83, CA 19–9, 17–1-A, C54–0, KL-1 for pancreatic adenocarcinoma [157, 172]. There is no data related to endometrial and ovarian cancers because the single cell analysis is still the method of choice.

We have used a use a newly employed marker gene, expressed in all cancer cells and not detectable in any non-tumor cells, the MMP-7 [173].

This gene plays a crucial role in tumor invasion and metastatic capacity. MMP-7 is not generally expressed in normal differentiated epithelial colon cells, but is found up regulated in CCR cells where is investigated for its metastatic potentiality. Up-regulation of MMP-7 occurs very early in colonic epithelial cells and has been found in 85% of colorectal adenocarcinomas and associated with a poor prognosis. In this series, MMP-7 is expressed in all patients who had relapsed, thus showing a sensitivity of 100%. The specificity is far lower (49%) and this is the major drawback of the methodic in use, because the test doesn't discriminate between colorectal and other cells such us mesothelium cells. However, cytology and immunochemistry could be helpful in differentiating colonic cells from others. The bioinformatics data highlighted the efficacy of MMP7 to specifically subselect tumours with higher aggressiveness, suggesting a potential role of negative prognostic marker. In addition strikingly MMP-7 appeared correlated to tgfb- 1. The correlation might probably potentiate the metastatic propensity of the cells, thus affecting progression of the disease and survival expectation. However, additional studies will be required to assess the cause/effect relationship between these two factors.

In conclusion, positivity of MMP-7 in peritoneal cavity samples could be a novel biomarker for predicting disease recurrence in patients with CRC.

## MATERIALS AND METHODS

### Patients and preparation of peritoneal washing

Between November 2009 and November 2011, all the patients undergoing elective resection for cancer of the colon or intra-peritoneal rectum were enrolled in this prospective longitudinal study. Exclusion criteria were preoperative chemo- o radiotherapy and cancer of the low rectum.

### Procedure

Peritoneal washing is done just after the laparotomy or laparoscopy, before colonic mobilization. One-hundred twenty ml of saline solution at 20° C is instilled at the site of the tumor and 60 ml of this fluid is then aspirated and collected in sterile containers and sent for RNA extraction. Samples are centrifuged at 670 G for 10 minutes. RNA is extracted from the cell precipitate using the TRIzol reagent (Invitrogen), reverse transcribed into complementary DNA (cDNA) and amplified by Real-time PCR using the following conditions: denaturation, 1 minute at 95°C; hybridization, 30 seconds at 50°C; and finally elongation of the filament at 75°C for 30 seconds. During PCR, temperature variations are used to control the activity of the polymerase and the binding of the primers. The sequence of the primers for the MMP-7 is FWD: 5′-ATGAACGCTGGACGGATGGT-3; REV: 5′-TGGAGTGGAGGAACAGTGCT-3′. The sequence of the primers for β-actin, used as an internal control, is: FWD: 5′-AAGATGACCCAGATCATGTTTGAG ACC-3; REV: 5′-AGCCAGTCCAGACGC AGGAT-3. The entire method is performed using the ‘IQ SYBR Green Supermix’ (Bio-Rad Laboratories, Milan). Each sample was analyzed in duplicate by Real-time PCR. A sample was considered positive for the gene analyzed when adequate amplification curve was detected in the dual evaluation conducted on the same sample. Samples were considered negative when no amplified or only one amplified was detected. This method, based on duplicate analysis, allows reducing to a negligible value the probability that the amplified does not correspond in fact to the gene under study.

### Follow-up

After surgical resection, all patients are undergoing regular follow-up for assessing recurrence. Postoperative surveillance includes onco-markers, CT scan and colonoscopy.

### Bioinformatic

Gene expression datasets were analysed by using oncomine online tool (onocomine.org). Analysis of Kaplan-Maier estimation curve has been performed as previously described [174, 175]. Briefly, samples were divided in two cohorts so that to maximize positive correlation between expression profiles of MMP7 and TGFb1. The separation of patients into “cohort 1” and “cohort 2” along with survival information is next used to find any statistical differences in survival outcome. The R statistical package is used to perform survival analyses^1^ and to draw KMplots.
